# Factors associated with perception of risk of contracting HIV among secondary school female learners in Mbonge subdivision of rural Cameroon

**DOI:** 10.11604/pamj.2014.17.259.2772

**Published:** 2014-04-08

**Authors:** Elvis Enowbeyang Tarkang

**Affiliations:** 1 HIV/AIDS Prevention Research Network, Cameroon (HIVPREC), Kumba, Southwest Region, Cameroon

**Keywords:** Perception of risk, secondary school female learners, Health Belief Model (HBM), HIV/AIDS, Rural Cameroon, Mbonge subdivision

## Abstract

**Introduction:**

Since learners in secondary schools fall within the age group hardest hit by HIV/AIDS, it is obvious that these learners might be at high risk of contracting HIV/AIDS. However, little has been explored on the perception of risk of contracting HIV among secondary school learners in Cameroon. This study aimed at examining the perception of risk of contracting HIV among secondary school learners in Mbonge subdivision of rural Cameroon using the Health Belief Model (HBM) as framework.

**Methods:**

A quantitative, correlational design was adopted, using a self-administered questionnaire to collect data from 210 female learners selected through disproportional, stratified, simple random sampling technique, from three participating senior secondary schools. Statistics were calculated using SPSS version 20 software program.

**Results:**

Only 39.4% of the respondents perceived themselves to be at high risk of contracting HIV, though the majority, 54.0% were sexually active. Multinomial logistic regression analyses show that sexual risk behaviours (p=0.000) and the Integrated Value Mapping (IVM) of the perception components of the HBM are the most significant factors associated with perception of risk of contracting HIV at the level p<0.05.

**Conclusion:**

The findings of this study can play an instrumental role in the development of effective preventive and interventional messages for adolescents in Cameroon.

## Introduction

The global HIV/AIDS situation for adolescents is extremely serious, and the need for stronger focused response is urgent. Young people are particularly vulnerable to HIV infection because of risky sexual behaviours and substance abuse. These are convoluted by lack of access to accurate and personalised HIV information and prevention services and a host of other socio-economic reasons [1].

The adoption of preventive behaviours would be the only protection against HIV transmission, if a major medical break-through stays a long way off. Despite an almost universal awareness of AIDS and the lethality of HIV sexual transmission, there is no correspondence with a widespread adoption of preventive measures. The socio-psychological literature on health-related behaviours emphasises the perception of being at risk of infection as one of the necessary conditions for behavioural change. Moreover, the degree of perceived risk seems to affect individuals’ actual control in adopting preventive measures [2].

In order for young people to take precautions to protect themselves against HIV infection, they first have to regard themselves as potentially at risk of becoming infected. Individuals who deny the presence of HIV/AIDS in their community have a perceived invulnerability to the disease. Individuals who have had sex should have a higher perceived HIV/AIDS risk than virgins; and individuals who have high-risk sex (no or infrequent condom use, or multiple partners) should have higher perceived risks than individuals who engage in low-risk sex [3].

Despite increasing awareness of the severity of HIV, youths in Cameroon are still engaging in risky sexual behaviours such as multiple sexual partners, and non-use of condoms [4, 5]. Risk perception of HIV/AIDS should accompany risky sexual behaviour change. But this is not always the case.

In 2013, which is now 32 years since AIDS was first discovered, Cameroon continues to be affected by the HIV/AIDS epidemic, despite increased levels of awareness of the disease. Recent estimate of prevalence level of HIV/AIDS in Cameroon is 5.3% [6].

Heterosexual transmission of HIV accounts for about 90% of new infections in Cameroon [7]. Hence young people are at risk of getting the disease as soon as they initiate sexual activity. In Cameroon, 61% of PLWHA are women [8], and the prevalence among women of reproductive age was 6.8% [9]. HIV/AIDS prevalence among females in villages in rural Cameroon is high, 6.3% [10]. This can be attributed to the fact that rural Cameroonians often face lack of job opportunities, declining incomes, bad educational and medical infrastructures and poverty which may lead to low perception of HIV infection risk.

Although rural settings comprise the majority of the country's population and in certain rural areas such as in the Southwest region where this study is conducted HIV prevalence is more than the national rate, 11.0% [11], little data on perception of HIV infection risk and the factors that influence perception of risk exists.

The purpose of this study is to understand the perception of risk of contracting HIV and factors associated with risk perception among secondary school female learners in Mbonge subdivision of rural Cameroon.

Knowing about female adolescents’ perceptions of risk of contracting HIV is important to understand why they engage in the behaviours they do, and this knowledge can play an instrumental role in the development of effective preventive and interventional messages.

Health professionals have been inquiring for years about how behavioural change occurs. Throughout literature the Health Belief Model (HBM) has been a commonly cited theory for behaviour change that acknowledges the importance of perceived risk in behaviour change. It posits that people who perceive themselves to be at risk of a negative outcome are more likely to reduce their risk behaviours than those who do not see themselves at risk [12].

In terms of the HBM's root in value-expectancy theory, attitudes are developed and modified based on assessments about beliefs and values [13]. Sensitivity to risk depends on factors other than knowledge of infection mechanisms; it also depends on behaviours, such as individual awareness of the illness (its prevalence, the severity of its symptoms, its lethality) and the perception of the general health status [14]. So perception of risk is explained by the components of the HBM.

This paper uses the main psychosocial concepts of the HBM, namely: perceived susceptibility, perceived severity, perceived benefit, perceived barriers, perceived self-efficacy and socio-demographic variables as the theoretical perspective to examine, explain and predict factors associated with perception of risk of contracting HIV among secondary school female learners in Mbonge rural area of Cameroon [15, 16].

## Methods

A quantitative, correlational design was adopted in this study. In this study, the accessible population included all the secondary school learners in Mbonge rural town of Cameroon; that portion of the target population to which the researcher had reasonable access [17].

A disproportional, stratified, simple random sample was selected for this study. Probability sampling was used because it increased the likelihood that all the elements in the population would have an equal chance of being included in the sample [18]. The school attendance registers of the learners were used as the sampling frame to select a sample of 210 grade 10 to grade 12 (Form five to upper sixth) female learners from three senior secondary schools in Mbonge subdivision of Cameroon.

The data were collected during the first term of 2012 by means of a self-administered questionnaire comprising items regarding socio-demographic characteristics, and items relating to the major components of the HBM, sexual behaviours and perception of risk of contracting HIV. A four-point Likert type scale was used to rate the responses, using the following response categories: strongly agree, agree, disagree and strongly disagree [19]. Strongly agree and agree were coded as the index category.

The questionnaire was pretested to clarify instructions, relevancy, usability and completion time, to refine and introduce modifications where necessary and to ascertain reliability and validity [20]. During the pre-test, 10 learners, who did not participate in the actual study, completed the questionnaires. They required no assistance, understood the questions and needed approximately 15 minutes to complete the questionnaires.

The final questionnaires were administered to 210 female learners from three senior secondary schools in Mbonge sub division of Cameroon during normal class periods with the permission of the principals and the co-operation of the teachers concerned. One research assistant was available to assist the learners and to answer questions while they completed the questionnaires.

The reliability of the research instrument used for the study was tested using the coefficient alpha and by pre-testing the questionnaires. The following types of validity were also established: face validity, content validity, construct validity and criterion-related validity. This was ensured by constructing items to represent the different components of the HBM, based on literature review. The questionnaires were also subjected to scrutiny by a statistician.

Permission to conduct this study was granted by the HIV/AIDS prevention Research Network, Cameroon (HIVPREC) an NGO for the prevention of HIV/AIDS through formalized education, working in the South West region of Cameroon, and the principals of the three participating schools. Participation was voluntary and informed written consent was obtained from each learner and her parents/guardians prior to data collection. A questionnaire was handed to each learner when she produced a signed consent form from a parent/guardian and from herself.

Anonymously completed questionnaires were kept in a separate container from the signed informed consent forms in order to maintain anonymity. Anonymity was also maintained by reporting the findings of the three schools combined and by not providing comparisons among the three schools. Confidentiality was maintained because only the researcher had access to the completed questionnaires, which were locked up. Subsequent to the acceptance of the research report, these would be destroyed.

Data were analysed using SPSS version 20. Data were summarized by means of descriptive statistics including the frequency table. More advanced statistics included the chi square test at the 0.05 significant level and the multinomial logistic regression test.

### Model Specification and Estimation Procedure

Multinomial logistic regression analysis was performed to examine the factors influencing the outcome variable “the perception of risk of contracting HIV”. Logistic regression does not require the predictors to be normally distributed, linearly related, or to have equal variances within each group. Logistic regression is especially useful when the distribution of responses on the dependent variable is expected to be non-linear with one or more of the independent variables [21, 22]. The procedure gives rise to estimates of the likelihood of a certain event occurring, given a set of explanatory variables.

The HBM was tested drawing on it relevant theory and assumptions with regard to this study. The aim was to retain the assumptions of the model's application as much as possible and to assess the contributions of each component of the HBM and the various combinations of the components with regard to perception of HIV infection risk among secondary school female learners in rural Cameroon.

The different modelling alternatives considered are: maintaining the assumptions of each component of the HBM; integration of the components of the HBM (Integrated Value Mapping (IVM)).

Model estimation focused on mapping out the significant drivers of the outcome variable “perception of risk of contracting HIV” from a vector of consistently significant components suggested by the relevant theory underpinning the HBM.

During the regression analyses, items under each component of the HBM were considered together. The dependent variable ‘perception of risk of contracting HIV’ remained the same for all the modelling alternatives (the major components of the HBM, sexual risk behaviours and the integrated value mapping (IVM)). For specific values of the independent variables (the various components of the HBM, sexual risk behaviours and the IVM), the estimated value of P is the probability of the event that respondents mentioned that they were at high risk of contracting HIV/AIDS.

### Measures

**Outcome (dependent) variable:** The outcome variable for this study is perception of risk of contracting HIV as reported by the female learners. This measure was derived from the question: “How at risk of contracting HIV are you?” The response options were: 4=High risk, 3=Moderate risk, 2=Small risk and 1=Not at risk. High risk is the index category.

### Explanatory (independent) variables

**Perceived susceptibility to HIV:** This was constructed from two questions, each considered separately: ‘HIV/AIDS is a serious threat in Cameroon’ and ‘A healthy looking person can be HIV positive’. The coefficient alpha for this 2-item scale was 0.418. The response options were rated on a four-point Likert scale as ‘3=strongly agree’, ‘2=agree’, ‘1=disagree’ and ‘0=strongly disagree’. ‘Strongly agree’ and ‘agree’ were coded as the index category.

**Perceived severity of HIV/AIDS:** This measure was based on the degree of agreement with the following statements: ‘HIV/AIDS is a disease like any other’, ‘Some traditional healers can cure AIDS’, ‘Some antibiotic can cure AIDS’ and ‘Anti-Retroviral Therapy (ART) can cure AIDS’. The Cronbach's alpha for this 4-item scale was 0.474. The response options were the same as for ‘perceived susceptibility’ and were coded in the same manner.

**Perceived Benefit Of Condom Use:** This measure was based on the degree of agreement with the following statement: ‘Correct and consistent use of condoms during sexual intercourse could prevent transmission of HIV/AIDS’. The response options were the same as for ‘perceived severity’ and were coded in the same manner.

**Perceived Condom Use Self-Efficacy:** This measure was based on the degree of agreement with the following statements: ‘I have confidence that I could refuse sex with my partner if he refuses to use condoms’ and ‘I feel confident that I can convince my partner(s) to use condoms during sexual intercourse’. This 2-item scale had a Cronbach's alpha of 0.572. The response options were the same as for ‘perceived benefit’ and were coded in the same manner.

**Perceived Barriers To Condom Use:** This measure was based on the degree of agreement with the following statements: ‘Should a condom slip off during sexual intercourse it will land up in my stomach’, ‘Latex condoms cause itching’, ‘I am allergic to lubricants used in condoms’ and ‘I feel embarrassed to ask my partner to use condoms’. The Cronbach's alpha for this 4-item scale was 0.499. The response options were the same as for ‘perceived self-efficacy and were coded in same manner.

**Socio-Demographic Variables:** The following socio-demographic variables were included in the study: age group, marital status, academic profile, house of residence, religious affiliation, and father's and mother's monthly incomes. Age was self-reported by respondents in years. Marital status was dichotomized as ‘single’ (index category) and ‘married or cohabiting’. Academic profile was dichotomized as ‘passed on merit’ (index category) and ‘promoted on trial or repeated’. House of residence was dichotomized as ‘5 rooms or more’ (index category) and ‘four rooms or less’. Religious affiliation was dichotomized as ‘Christian’ (index category) and ‘others’. Father's and mother's monthly incomes were dichotomized as ‘200 000XAF and above’ (index category) and ‘less than 200 000XAF’.

**Sexual Experience:**This was measured with the question: Have you ever had sexual intercourse with a male partner? With ‘1=yes’ or ‘0=no’ as response options.

**Sexual risk behaviors:** The following sexual risk behaviours were included in this study: number of sexual partners and condom use. Number of sexual partners was categorised as ‘more than one’ (index category), ‘one’ and ‘none’. Condom use during first and last sexual encounters were dichotomized as ‘yes’ (index category) and ‘no’, while regularity of condom use was categorized as ‘1=always’, ‘2=most of the time’, ‘3=seldom’ and ‘4=never’. These questions were asked only to respondents who were sexually active.

## Results

### Descriptive statistics

The descriptive statistics of the explanatory and dependent variables are shown in Table 1. Most of the respondents (92.4%; n=194) were 16-24 years old. Most respondents (93.3%; n=194) were single, and all were senior secondary school female learners. Of the respondents, 98.0% were Christians. Most of the respondents (72.5%) passed their exams on merit. Most of their fathers’ and mothers’ monthly incomes were less than 200 000XAF (51.7% and 76.2%) respectively. With respect to the different components of the HBM, perceived susceptibility to HIV/AIDS was quite high, perceived severity of HIV/AIDS was also high and perceived effectiveness of using condoms to prevent HIV/AIDS was relatively high. Relatively fewer respondents perceived some barriers to condom use, while perceived self-efficacy for condom use was also high. Regarding the perception of risk of contracting HIV/AIDS, only 39.4% indicated that they were at high risk of HIV infection, although 54.0% were sexually active.

**Table 1:** Characteristics of secondary school female learners in Mbonge, Cameroon

Characteristic	Frequency	Percentage
**Age group**		
11-15	16/210	7.6
16-24	194/210	92.4
**Marital status**		
Single	194/208	93.3
Married or cohabiting	14/208	6.7
**Academic profile**		
Pass on merit	150/207	72.5
Promoted on trial or repeated	57/207	27.5
House of residence		
5 rooms or more	107/203	52.7
4 rooms or less	96/203	47.3
**Religious Affiliation**		
Christian	195/199	98.0
Others	4/199	2.0
**Father's monthly income (in XAF)**		
200 000 and above	85/176	48.3
Less than 200 000	91/176	51.7
**Mother's monthly income (in XAF)**		
200 000 and above	50/185	23.8
Less than 200 000	135/185	76.2
Perceived susceptibility to HIV/AIDS		
**HIV/AIDS is a serious threat in Cameroon**		
Agree	154/198	77.7
Disagree	44/198	22.3
**A healthy looking person can be HIV positive**		
Agree	180/205	87.8
Disagree	25/205	12.2
**Perceived severity of HIV/AIDS**		
**HIV/AIDS is a disease like any other**		
Agree	108/198	54.5
Disagree	90/198	45.5
**Some traditional healers can cure AIDS**		
Agree	29/204	14.3
Disagree	175/204	85.7
**Some antibiotics can cure AIDS**		
Agree	31/198	15.6
Disagree	167/198	84.4
**Anti-Retroviral Therapy (ART) can cure AIDS**		
Agree	37/197	18.8
Disagree	160/197	81.2
Perceived benefit of condom use		
Correct and consistent condom use can prevent HIV/AIDS		
Agree	153/197	77.6
Disagree	44/197	22.4
**Perceived barriers to condom use**		
**Should a condom slip off during sex it will land up in my stomach**		
Agree	58/179	32.4
Disagree	121/179	67.6
**Latex condoms cause itching**		
Agree	74/139	53.2
Disagree	65/139	46.8
**I am allergic to lubricants used in condoms**		
Agree	50/138	36.2
Disagree	88/138	63.8
**I feel embarrassed to ask my partner to use condoms**		
Agree	73/186	39.2
Disagree	113/186	60.8
Perceived condom use self-efficacy		
**I have confidence that I could refuse sex with my partner if he refuses to use condoms**.		
Agree	147/196	75.0
Disagree	49/196	25.0
**I feel confident that I can convince my partner(s) to use condoms during sexual intercourse**.		
Agree	130/192	67.7
Disagree	62/192	32.3
**Perception of risk of contracting HIV/AIDS**		
**How at risk of contracting HIV/AIDS are you?**		
Not at risk	89/203	43.8
Small risk	18/203	8.9
Moderate risk	16/203	7.9
High risk	80/203	39.4
**Sexual experience**		
**Have you ever had sexual intercourse with a male partner?**		
Yes	108/200	54.0
No	92/200	46.0
**Number of sexual partners in the past one year**		
**How many sexual partners have you had in the past one year?**		
None	12/97	12.4
One	54/97	55.6
More than one	31/97	32.0
**Number of concurrent sexual partners**		
**How many sexual partners do you have at present?**		
None	20/97	20.6
One	68/97	70.1
More than one	9/97	9.3
**Condom use during first sexual encounter**		
**Did you use a condom the first time you had sexual intercourse with a male partner?**		
Yes	41/103	39.8
No	62/103	60.2
**Condom use during last sexual encounter**		
**Did you use a condom the last time you had sexual intercourse with a male partner?**		
Yes	52/105	49.5
No	53/105	50.5
**Regularity of condom use**		
**How often do you use a condom with a sexual partner during sex?**		
Always	32/108	29.6
Most of the time	33/108	30.6
Seldom	14/108	13.0
Never	29/108	26.8
Denominators may vary due to missing values		

### Model result

The level of significance of the various components of the HBM is explained by the P values of the log-likelihood chi-square statistics (Table 2). If this P value is discussed at alpha=0.05, then none of the components of the HBM have good significant levels therefore denoting the inadequate explanatory power of each of these components in explaining perception of HIV infection risk among female learners in rural Cameroon. However, according to the Pseudo-R Square (Cox and Snell) values, these components explained between 4-45% of variation in the perception of HIV infection risk.

**Table 2:** Multinomial logistic regressions between explanatory variables and perception of risk of contracting HIV/AIDS

No	Model components	LR Chi-Square	df	P values	Pseudo R-Square	N	Explanatory power of model
1	Perceived susceptibility to HIV/AIDS.	13.782	18	0.743	0.070	190	7.0%
2	Perceived severity of HIV/AIDS.	39.256	36	0.326	0.191	185	19.1%
3	Perceived Benefit of condom use.	7.668	9	0.568	0.039	193	3.9%
4	Perceived Barriers to condom use.	46.300	36	0.117	0.327	117	32.7%
5	Perceived condom use self-efficacy.	16.639	18	0.548	0.085	187	8.5%
6	Socio-Demographic variables.	89.919	75	0.115	0.447	152	44.7%
7	Sexual risk behaviors	85.553	45	0.000	0.365	184	36.5%
8	Integrated value mapping (IVM): combination of components 1, 2, 3 and 6.	203.305	138	0.000	0.816	120	81.6%
9	Integrated value mapping (IVM): combination of components 1, 2, 3, 4 and 5.	161.060	117	0.004	0.800	100	80.0%

The IVM for perceived susceptibility to HIV/AIDS, perceived severity of HIV/AIDS, perceived benefit of condom use and the socio-demographic variables remained very stable, with P=0.000 (Table 2). This IVM had the strongest explanatory power, 81.6% (Pseudo R-Square=0.816).

After controlling for the socio-demographic variables, the IVM for the perception components of the HBM was also significant, p=0.004, with an explanatory power of 80.0% (Pseudo R-Square=0.800).

Sexual risk behaviours also had a good significant level, p=0.000, with an explanatory power of 36.5% (Pseudo R-Square=0.365), denoting the adequate explanatory power of sexual risk behaviours in explaining perception of risk of HIV infection among female learners in rural Cameroon.

The likelihood ratio test (Table 3) summarises the relationship between the predictors and the outcome variables for sexual risk behaviours and the IVMs.

**Table 3:** Components of the Integrated Value Mapping and sexual risk behaviours: Likelihood Ratio Tests

Effect	-2 Log Likelihood of Reduced Model	Chi-Square	df	Sig.
Perceived susceptibility to HIV/AIDS				
HIV/AIDS is a serious threat in Cameroon	70.677	66.775	12	0.000
Perceived severity of HIV/AIDS				
HIV/AIDS is a disease like any other	117.810	32.868	9	0.000
Perceived barrier condom use				
I feel embarrassed to ask my partner(s) to use condoms during sex	186.687	17.589	9	0.040
Socio-demographic variables				
House of residence	129.089	44.147	12	0.000
Religious affiliation	125.605	40.668	15	0.000
Sexual risk behaviours				
Number of sexual partners at present	161.194	21.399	9	0.011
Did you use a condom the first time you had sexual intercourse with a male partner?	155.110	15.315	6	0.018
How often do you use a condom with a male partner during sexual intercourse?	168.789	28.994	12	0.004

The results as depicted in Table 3 reveal that learners who did not perceive that HIV/AIDS is a serious threat in Cameroon, who perceived HIV as a disease like any other, who had multiple concurrent sexual partners, who did not use condoms during their first sexual encounters, who used condoms inconsistently, who felt embarrassed to ask their partners to use condoms during sex, who live in high income houses and who are Christians, are more likely to perceive low risk of contracting HIV/AIDS.

## Discussion

The majority of the respondents were among the age group hardest hit by HIV/AIDS [23]. Single persons are predisposed to sexual temptations which might increase their vulnerability to STIs and HIV/AIDS [24]. Women and young girls lack power over their bodies, and their sexual lives, social and economic inequalities increase their vulnerability for contracting and living with HIV/AIDS. With increasing levels of poverty in Cameroon, women especially female learners have found themselves in casual relationships with men for financial gains. Women might therefore find it difficult to demand condom use, as they become subordinates or dependent of mainly older men; women are also biologically prone to infection, and HIV is more easily transmitted from men to women than the reverse [25].

Religion could hamper the effective use of condoms for HIV prevention [26]. The Roman Catholic Church opposes condom use in favour of “direct contact” [27, 28]. This could have serious implications for spreading HIV.

A high level of academic engagement has an influence on the age of sexual initiation and makes health education messages more meaningful [29, 30]. Adolescents with high academic aspirations are more likely not to jeopardize their academic careers by unwanted pregnancies, and STDs, including HIV/AIDS, by not using condoms.

Table 1 reveals that only 39.4% of the respondents perceived themselves at high risk of HIV infection. This percentage is higher than that obtained by Pettifor et al in South Africa (14.0%) [31], but lower than that obtained by Munthali et al in Malawi, (79.0%) [32]. This disparity could be due to the cultural differences in relation to sexual activities between these two countries and Cameroon.

Young people often perceive their risks of HIV/AIDS to be low even if they engage in HIV/AIDS risky behaviours, live in areas with high HIV prevalence rates, or are knowledgeable about HIV/AIDS. One explanation for low perceived HIV/AIDS risk is that youths may exhibit optimistic bias, tending to underestimate risks in general due to feelings of invulnerability [33, 34]. Additionally, HIV/AIDS is a highly stigmatised disease. Acknowledging one's own risk implies putting oneself at risk of being stigmatised. Thus youths may avoid self-disclosure, this by downplaying their own personal risk, which leads to further low risk perceptions [33]. Personality experience and familiarity with HIV/AIDS may be associated with more awareness of infection pathways, less stigma towards the disease, and higher perceived risks of infection. It is the actual perception of risk by the individuals that matters in their decision making, not whether that perception is known to be correct or incorrect.

In accordance with other studies in Cameroon and SSA this study have found correlations between perceived risk of contracting HIV/AIDS and sexual risk behaviours [35–38]. However this study contradicts earlier findings by Tarkang et al, in Kumba, Cameroon in 2011, who reported perceived barriers to condom use and socio-demographic variables as the major factors influencing perception of HIV infection risk [39]. This disparity could be due to the different settings of the two studies. The 2011 study was carried out in an urban setting, while this study was performed in a rural setting.

This study demonstrated the utilization of the HBM for investigation factors associated with perception of risk of contracting HIV among senior secondary school female learners in Mbonge subdivision of Cameroon.

The concepts and relationships described within the HBM work synergistically to create a greater understanding of the phenomenon of interest, reducing or avoiding a disease condition (HIV/AIDS) and an aim to explain or predict health behaviours [40].

The HBM has the premise that individuals will take action to prevent, control or treat a health problem if they perceive themselves to be susceptible to the health problem, if they perceive the problem to be severe in its nature and/or in its consequences, if they perceive that the action will benefit them and produce desirable outcomes, if they perceived few barriers to taking that action and if they believe in their ability to successfully take the recommended action to prevent, control or treat the health problem [12].

The log likelihood ratio tests suggest that the items in Table 3 with a significance level of p<0.05, were the most significant factors associated with perception of risk of contracting HIV/AIDS. All these items should be considered in designing any policy geared towards increasing perception of risk of HIV infection among female learners in Mbonge, Cameroon.

The important point this study brings to the fore is that the integration of the perception components of HBM and sexual risk behaviours are the main factors associated with perception of risk of contracting HIV among female students in rural Cameroon.

The findings also implied that the integration of these perception components resulted in a synergistic effect on perception of HIV infection risk. Our findings suggest that HIV prevention programmes for female students should emphasise these components concurrently as well as sexual risk behaviours as a strategy to improve on the perception of HIV infection risk.

As depicted by the results of this study, health education messages that focus on the components of the HBM in isolation as a strategy to increase the perception of risk of contracting HIV among female learners in rural Cameroon may be counterproductive.

Given the vulnerability of young women to HIV, it is of program and policy relevance to better understand the relationship between actual behavioural risk and perceptions of risk among secondary school female learners in Mbonge subdivision of rural Cameroon in order to help them protect themselves from negative outcomes. The ability to accurately judge one's risk to HIV is an essential element in developing successful strategies for prevention.

If people are not aware of the level of risk that their activities involve, knowledge on preventive measures as such will not reduce the risks taken. The degree of riskiness of unprotected sex is not often addressed in HIV prevention campaigns; and neither are evaluations on the relation between risk perception and preventive behaviour widely incorporated in the empirical research on condom use.

## Conclusion

Although further investigation is still needed, the overall impression is that the study justifies the HBM as a useful model in understanding, explaining and predicting perception of risk of contracting HIV/AIDS among female students in rural Cameroon. The integration of perception components of the HBM working in synergy and sexual risk behaviours are important and direct factors influencing perception of risk of HIV infection among female learners in Mbonge subdivision of Cameroon.

### Limitation

This study has several limitations. The internal consistency of some of the HBM components was relatively low. Since the value of the Cronbach's alpha depends on the inter-correlation and the number of items [41], the low Cronbach's alpha can be explained by the heterogeneity in HIV-related behaviours and the small number of items within each component of the HBM. In addition, because most of the items in the questionnaire elicit self-reported information on sensitive issues such as condom use and HIV/AIDS, the respondents might have been bias in responding to these items. However assurance of confidentiality and anonymity might have minimized this problem.
